# Assessing Very Early Infant Diagnosis Turnaround Times: Findings from a Birth Testing Pilot in Lesotho

**DOI:** 10.1155/2017/2572594

**Published:** 2017-12-19

**Authors:** Michelle M. Gill, Heather J. Hoffman, Majoalane Mokone, Vincent J. Tukei, Matsepeli Nchephe, Mamakhetha Phalatse, Appolinaire Tiam, Laura Guay, Lynne Mofenson

**Affiliations:** ^1^Elizabeth Glaser Pediatric AIDS Foundation, Project SOAR (Supporting Operational AIDS Research), Washington, DC, USA; ^2^Department of Epidemiology and Biostatistics, Milken Institute School of Public Health, George Washington University, Washington, DC, USA; ^3^Elizabeth Glaser Pediatric AIDS Foundation, Project SOAR (Supporting Operational AIDS Research), Maseru, Lesotho; ^4^Ministry of Health, Maseru, Lesotho; ^5^Centre for International Health, University of Bergen, Bergen, Norway

## Abstract

Very early infant diagnosis (VEID) (testing within two weeks of life), combined with rapid treatment initiation, could reduce early infant mortality. Our study evaluated turnaround time (TAT) to receipt of infants' HIV test results and ART initiation if HIV-infected, with and without birth testing availability. Data from facility records and national databases were collected for 12 facilities offering VEID, as part of an observational prospective cohort study, and 10 noncohort facilities. HIV-exposed infants born in January–June 2016 and any cohort infant diagnosed as HIV-infected at birth or six weeks were included. The median TAT from blood draw to caregiver result receipt was 76.5 days at birth and 63 and 70 days at six weeks at cohort and noncohort facilities, respectively. HIV-exposed infants tested at birth were approximately one month younger when their caregivers received results versus those tested at six weeks. Infants diagnosed at birth initiated ART about two months earlier (median 6.4 weeks old) than those identified at six weeks (median 14.8 weeks). However, the long TAT for testing at both birth and six weeks illustrates the prolonged process for specimen transport and result return that could compromise the effectiveness of adding VEID to existing overburdened EID systems.

## 1. Introduction

Without antiretroviral therapy (ART), infants with perinatal (in utero or intrapartum) HIV infection experience rapid clinical deterioration and high mortality [1]. In the absence of treatment, an early peak in mortality as high as 20–30% occurs among perinatally infected infants between 8 and 12 weeks of age, increasing to 68% by their second birthday [1–4]. However, early infant diagnosis (EID) of HIV followed by prompt initiation of ART can dramatically reduce mortality and morbidity among HIV-infected infants. Initiating ART in HIV-infected infants between 6 and 12 weeks of age has been shown to reduce early infant mortality by 76% and to decrease the progression of HIV disease by 75% [5].

The 2013 World Health Organization (WHO) recommendation for EID in resource-limited settings includes virologic testing of HIV-exposed infants using an HIV nucleic acid polymerase chain reaction (PCR) assay at four to six weeks of age or at the earliest opportunity thereafter [6]. However, only half of HIV-exposed infants (HEI) in the 21 African Global Plan countries received EID within two months of life [7]. Moreover, the WHO recommendation may contribute to delayed diagnosis among young infants, particularly those with in utero infection. By six weeks of age, many HIV-infected infants will have already become immunologically suppressed, while others may have died from HIV-related causes before even accessing EID [8–10]. A recent study in South Africa showed that 62% of infants had advanced HIV disease (defined as CD4 < 25% or WHO clinical stage 3 or 4) before starting ART at a median age of only 8.4 weeks [11].

Conventional sub-Saharan African EID programs involve collection of dried blood spot (DBS) samples from infants at clinics, transport of the DBS sample to central laboratories for DNA-PCR testing, and return of paper results through the same transport network, with results returned to caregivers at their next clinic visit. Prolonged turnaround times (TAT), sample transport difficulties, and supply chain management limitations result in high loss to follow-up before ART initiation, with only about 50% of infants tested ever receiving their test results [12–14]. With EID at six weeks of age, ART initiation among infants retained in care may not occur until three to four months of age or older—after the initial peak in mortality and the recommended period of early ART initiation [15].

Very early infant diagnosis (VEID), defined as testing within two weeks of life, combined with rapid ART initiation could prevent the observed decline in immunologic function and clinical deterioration and further reduce infant mortality. In 2015, the South African government revised its prevention of mother-to-child transmission (PMTCT)/EID guidelines to include routine birth testing for all HIV-exposed neonates [16], while other countries are planning a similar adoption and the WHO guidelines include a consideration of this approach [17, 18]. While birth virologic testing appears to be a promising intervention, questions remain about how an additional test would be integrated into existing EID systems, including how long it would take to transport and test additional specimens and obtain results, particularly in limited resource settings with already overburdened laboratories. While TAT for six-week EID testing has been well-documented in the literature, there are limited data on TAT for birth testing.

We evaluated TAT for receipt of HIV test results for HEI tested at birth and six weeks and time to ART initiation for HIV-infected infants born to HIV-positive women in Lesotho from sites participating in an observational cohort study that included birth testing and additional sites conducting routine testing starting at six weeks.

## 2. Methods

### 2.1. Program and Study Cohort Description

Birth DNA-PCR testing was added prospectively to the standard EID testing algorithm at ages 6 and 14 weeks at 13 facilities in three districts of Lesotho. These facilities were participating in an observational prospective cohort study evaluating the effectiveness of PMTCT service delivery. DBS collection was conducted at birth and 6 and 14 weeks using similar procedures. Infant blood collection took place at delivery wards and maternal-child health (MCH) clinics at health centers and hospitals. If the district lab and MCH clinic were located on the same hospital campus, nurses would hand-deliver specimens to the district lab; for most health centers, specimens were transported to district laboratories via motorbike. Because district laboratories do not perform DNA-PCR testing, district laboratories registered specimens and compiled them with others, before transporting the samples to the national reference laboratory (NRL). Specimens were tested at NRL as they were received, without prioritizing samples based on date of blood draw. Printouts of test results generated by the laboratory information system were then returned to district laboratories and then transported back typically by motorbike to health centers. Cohort study nurses or HIV counselors delivered results to caregivers. Results were often delivered at the next postnatal visit for infants with HIV-negative test results. For infants with HIV-positive test results, MCH staff and community health workers contacted women by phone or home visit to invite them back to the clinic to receive the results.

### 2.2. Study Design

The study to evaluate test turnaround time took place in 12 out of 13 cohort facilities (excluding one which was no longer operational at the time of this study) and 10 noncohort facilities in three out of 10 districts of Lesotho. The three districts represented the geographical regions of the country: highlands (Thaba-Tseka), foothills (Butha-Buthe), and lowlands (Mohale's Hoek). All five hospitals in the three districts and seven medium- and high-volume lower level health centers that participated in the cohort were included in this study. All of the remaining 10 medium-volume health centers that conducted only standard-of-care six-week testing in the same districts were also included in the study (the noncohort facilities). We included noncohort facilities to determine if there was shorter TAT at cohort facilities due to the additional support from cohort study nurses who may have conducted more follow-up on results than what might typically be done.

All HIV-exposed infants in the 12 cohort facilities with documentation of a birth or six-week test and in the 10 noncohort facilities with documentation of a six-week test born between January and June 2016 were included in the analysis. A birth test was defined as testing before two weeks of life and a six-week test was defined as testing between four and nine weeks of age. Data were collected from July to September 2016 using paper-based forms. Trained study staff abstracted data from study and facility records related to infants' birth and six-week DNA-PCR tests including dates of blood draw, dates results were returned to the facility, dates results were returned to the caregiver, HIV test results, and dates of ART initiation (HIV-infected infants only). Additional data were extracted from national EID and laboratory databases, including dates the specimen was received and tested at the NRL. Infants' records were included even if their information was missing from the national database. Because information contained in the EID database was limited, any infants in the database who could not be located in facility records and verified with clinic staff were excluded.

Because so few HIV-infected infants were captured during the January–June 2016 timeframe, we also collected the same data for all live-born infants enrolled in the cohort who were diagnosed as infected at birth (*N* = 5) or six weeks (*N* = 2) from the start of the cohort in July 2014 to contribute additional data to the time to ART initiation outcome. All completed data were reviewed by the study coordinator before entry into an MS Access database.

### 2.3. Data Analysis

Total TAT from blood draw to caregiver result receipt or ART initiation for HIV-exposed and infected infants, respectively, was calculated. Infant age at the time the caregiver received the results or at the time of ART initiation and the length of time (in days) it took to complete interim steps along the V/EID pathway were also determined. Interim steps were as follows: (1) from blood draw to specimen receipt at NRL; (2) from receipt of specimen at NRL to testing at NRL; (3) from testing at NRL to result receipt at facility; (4) from result receipt at health facility to receipt by caregiver; and (5) from result receipt by caregiver to treatment initiation. Infants' data were included if dates were available to calculate at least one step; the number of infants contributing to each step varied as some dates were not available to calculate all steps for each infant. Using SAS/STAT software, descriptive statistics were calculated for all variables of interest using frequencies and percentages for categorical variables and medians (interquartile ranges) and minimum and maximum ranges for continuous variables. Wilcoxon rank sum tests were used to examine differences in TAT from blood draw to caregiver result receipt between cohort and noncohort facilities and TAT from blood draw to caregiver result receipt between infants tested at birth and infants tested at six weeks at study facilities. Differences in the number of days for the interim steps (1–4 above) between three groups (birth test/cohort facility, six-week test/cohort facility, and six-week test/noncohort facility) were examined with Kruskal-Wallis tests.

### 2.4. Ethical Considerations

Ethical approvals were received from the institutional review boards of the Lesotho Ministry of Health and the George Washington University. We received a waiver of informed consent. All study staff involved in data collection were trained in human subjects' research ethics and signed a research confidentiality agreement.

## 3. Results

Among the children born during January and June 2016, there was 1 HIV-infected child out of 83 HEI tested at birth. There was also 1 HIV-infected child out of 83 HEI tested at six weeks at cohort facilities and 4 HIV-infected children out of 87 HEI tested at six weeks at noncohort facilities. However, one HIV-infected child from a noncohort facility was excluded from this analysis due to insufficient data in the facility register to calculate steps 1–4 and the infant's date of ART initiation could not be verified. Therefore, data from only three HIV-infected infants are presented.

Among infants at cohort facilities, the median infant age at the time caregivers received birth HIV test results was 78 days (IQR 48–102) and 105.5 days (IQR 94–118) for infants tested at six weeks (Table 1). At noncohort facilities, infants were a median age of 111.5 days (IQR 104–121) when their caregivers received the six-week test results (*p* = 0.36 cohort versus noncohort facilities). Median TAT from blood draw to caregiver receipt of results was 76.5 days (IQR 48–98) at birth versus 63 days (IQR 52–72) at six weeks at cohort facilities; this difference was not significant (*p* = 0.12). TAT was 70 days (IQR 56–74) for the six-week test at noncohort facilities; the difference was not significant when compared to the TAT for the six-week test at cohort facilities (*p* = 0.42). The age of the infant at the time of caregiver result receipt for birth testing ranged from 32 to 174 days.

There were no significant differences between groups (birth/cohort; six-week/cohort; six-week/noncohort) for any of the interim steps. The time from receipt of the specimen at the NRL to testing at the NRL was the shortest of the interim steps in terms of the median number of days: seven, six, and six days for birth testing, six-week testing at cohort facilities, and six-week testing at noncohort facilities, respectively.

The TAT from blood draw to ART initiation was 38 days for the one cohort infant identified as infected at birth versus 49 days for the one cohort infant identified as infected at six weeks and 66 days for the one infected infant with available data at the noncohort facility (ART initiation dates for the other two infected infants were not available from existing facility records). The two infected cohort infants were initiated on ART the same day the caregiver received the results; the infected noncohort infant was initiated on ART 10 days after result receipt.

In the overall cohort study, of the 627 HEI born, a birth test was performed for 69.7% of infants (*n* = 437); test results of six infants were missing. Nine mothers (1.4%) declined birth testing and 22 infants (3.5%) died within the first hours or days of life. Other reasons for not having a birth test performed included home delivery, delivery over a weekend or other time without a study nurse on duty, delivery at a nonstudy facility, and failure to perform the test by a study nurse. Five children were identified as infected at birth and two children were identified as infected at six weeks (Table 2). One child at each of these time points was also captured in the first analysis as they were born between January and June 2016. One child infected at six weeks had a negative birth HIV test result and the other was not tested at birth, as the mother returned to the study facility after delivery but outside the two-week postpartum window for birth testing. The median TAT from blood draw to ART initiation was 38 days (IQR 35–48) for the five infants diagnosed as HIV-infected at birth with a range of 28 to 62 days. The median TAT from blood draw to ART initiation was 56 days (49 and 63 days) for the two HEI diagnosed at six weeks.

## 4. Discussion

Infant HIV status was known approximately one month earlier for HEI who were tested at birth (median age 78 days) compared to those who had a DNA-PCR test performed at six weeks (median 105 days and 111.5 days at cohort and noncohort facilities, resp.). For the few infants who were HIV-infected, the infants tested at birth were almost two months younger than those tested at six weeks at the time caregivers received the test results (median age 43 days for those tested at birth versus 97.5 and 94.5 days for those tested at six weeks in cohort and noncohort facilities, resp.). Receipt of HIV test results for HIV-infected infants was expedited. Because ART initiation occurred soon after being diagnosed as infected, the infants tested at birth also started on treatment at a younger age (45 days) than those tested at six weeks (104 and 112 days old at cohort and noncohort facilities, resp.). These are similar to data from Thailand, where testing one to two days after birth reduced infant age at HIV diagnosis from 78 days (for testing performed at four and eight weeks) to 36 days [17].

However, our findings also illustrate the prolonged process for specimen transport and results transfer for testing at both birth and six weeks. The median TAT from blood draw to caregiver receipt of results for HIV-exposed infants tested at birth and six weeks was over two months, which is largely unchanged from a prior study conducted on TAT in Lesotho based on data from 2011 [19]. Other countries have reported even longer TAT. For example, in rural Zambia, DNA-PCR results took approximately three months to be returned to caregivers of HIV-exposed infants [20]. The median age in which caregivers in our study received the birth test results was approximately 11 weeks, which meant infants often had blood drawn again at six weeks before their caregivers received results from the first test.

TAT from six-week blood draw to caregiver receipt of results was a week shorter at cohort versus noncohort facilities (although this was not statistically significantly different) and over two weeks longer at noncohort facilities from blood draw to ART initiation for the one HIV-infected infant with comparison data available. Generally, nurses at all facilities follow up with the NRL if test results were not received at the facility six weeks after testing, identify women who miss appointments (including those whose infants' HIV-negative test results would be available), and engage community workers to conduct tracing. However, cohort nurses may have been more persistent in their follow-up and had more resources to do so (e.g., airtime for making phone calls) leading to slightly shorter TAT at cohort facilities. For other steps along the pathway, such as transport to and from the NRL and testing at the NRL, it is less likely that participation in the cohort made any difference in time, as study samples were not prioritized over nonstudy samples for transportation or testing.

The short time between facility and caregiver receipt of results and ART initiation for HIV-infected infants at cohort facilities indicates that these children were being fast-tracked. All cohort infants identified as infected at birth were initiated on ART by approximately nine weeks of age. In a pilot program in a South African hospital, nearly all HIV-infected infants who were tested hours after birth were initiated on ART by two weeks of age [21]. However, timely communication to caregivers for negative results are also important; these results may offer psychosocial benefits to the family and could help to keep mother/infant pairs engaged in care [13, 22].

Approximately 1% (*N* = 5/431) of children enrolled in the cohort study who had a birth test result were diagnosed as infected at birth. The low transmission rate despite the high prevalence of HIV of 30% among childbearing-aged women in Lesotho reflects the implementation of Option B+ nationally in 2013; in 2015, 70% of HIV-positive pregnant women received ART [7, 23]. Lesotho has one of the highest six-week EID testing coverage rates, at 93% in 2015 [7]. However, among all the Global Plan countries, only half of infants received a virologic test at two months of age, suggesting there is considerable room for improving existing systems. One study evaluating the clinical impact and cost-effectiveness of different testing strategies in South Africa found that clinical outcomes improved more by scaling-up partially implemented six-week alone strategies than by adding birth testing; for example, improving coverage and result-return frequency from 50% to 100% improved one-year survival from 63.4% to 74.9%, while adding birth testing when six-week testing had 50% coverage and result-return improved survival minimally to 64.4%. [16].

One way to improve TAT and ensure more timely treatment initiation for infants identified as HIV-infected would be to decentralize testing to the district level or establish point-of-care (POC) HIV diagnostic machines [18]. This could be done with or without a policy shift to birth testing. While POC birth testing experience to date is limited, POC EID testing around 6 weeks in Mozambique demonstrated same-day TAT for results returned to caregivers compared to a median TAT of 122 days for samples sent to the reference laboratory [24]. Decentralized testing could also reduce the burden to a health system in which testing is conducted at a single national laboratory, though evidence from the CD4 POC diagnostics experience has highlighted some operational barriers to this approach [25].

Depending on the current in utero transmission rate and program effectiveness, strengthening of countries' existing six-week EID system should also be considered. Interventions to improve EID which have demonstrated success in reducing TAT include short message service (SMS) printers or SMS texts to relay results from testing laboratories to health facilities and use of geographical information systems to map health facilities to sample transport hubs within a certain distance and along road networks [13, 26, 27]. Ensuring these interventions are consistently implemented or functioning properly will be key to the success of system improvement. However, the potential reduction in early mortality in HIV-infected children with birth testing followed by immediate ART would be lost.

Our study had limitations. Since Lesotho has an effective PMTCT program, data abstraction yielded relatively few HIV-infected infants diagnosed at different time points. Additionally, incomplete existing records at noncohort study sites also limited the number of infants with complete TAT data. This resulted in reducing our overall modest sample size of 253 (for cohort and noncohort populations tested at birth and six weeks) to a lower number of infants contributing dates and limiting the conclusions that can be drawn regarding turnaround times, particularly with regard to comparisons between birth and six-week testing and cohort and noncohort facilities.

## 5. Conclusions

This study adds to the growing evidence on birth testing and helps to address the question of just how quickly infants could be initiated on treatment when a test at birth is added to the EID algorithm. While infants identified as infected at birth were initiated on treatment more quickly than those tested at six weeks, the TAT was still long and the findings highlight the key challenge of TAT for results within the EID system, which could be further exacerbated by national implementation of birth testing unless additional resources are provided to ensure testing capability is maintained. Country EID coverage, infant HIV transmission rates, and current clinic and laboratory capacity should be taken into consideration when devising the optimal approach to accelerate treatment initiation for infants identified as HIV-infected, thus minimizing HIV-associated morbidity and mortality.

## Figures and Tables

**Table 1:** Turnaround time (in days) for infants born and tested at cohort study facilities at birth and six weeks and infants born at noncohort study facilities tested at six weeks between January–June 2016.

	Blood draw to receipt at NRL^+^	Receipt of specimen at NRL to testing at NRL	Testing at NRL to result receipt at health facility	Result receipt at facility to receipt by caregiver	Blood draw to receipt by caregiver	Infant age at receipt by caregiver	Result receipt by caregiver to treatment initiation	Blood draw to ART initiation	Infant age at treatment initiation
*Birth (cohort study facilities only)*									
All infants, *N*^*∗*^,	40	48	43	60	54	61			
Median time (IQR)	22.5	7.0	15.0	14.5	76.5	78.0			
	(19.0–30.0)	(3.5–10.5)	(10.0–27.0)	(4.0–33.5)	(48.0–98.0)	(48.0–102.0)			
All infants, range	3.0–64.0	2.0–64.0	0–210.0	0–97.0	36.0–164	32.0–174.0			
HIV-infected infant (*n* = 1)	25.0	5.0	7.0	1.0	38.0	43.0	0	38.0	43.0

*6-week DNA-PCR (cohort study facilities)*									
All infants, *N*	44	53	48	58	46	58			
Median time (IQR)	21.0	6.0	15.5	13.0	63.0	105.5			
	(14.0–31.5)	(3.0–8.0)	(7.5–26.5)	(3.0–28.0)	(52.0–72.0)	(94.0–118.0)			
All infants, range	1.0–77.0	0–26.0	0–65.0	0–99.0	35.0–158.0	62.0–201.0			
HIV-infected infant (*n* = 1)	28.0	8.0	8.0	5.0	49.0	102.0	0	49.0	102.0

*6-week DNA-PCR (noncohort study facilities)*									
All infants, *N*	49	37	18	27	30	30			
Median time (IQR)	17.0	6.0	15.0	17.0	70.0	111.5			
	(11.0–27.0)	(4.0–9.0)	(8.0–21.0)	(8.0–31.0)	(56.0–74.0)	(104.0–121.0)			
All infants, range	0–57.0	0–78.0	7.0–66.0	0–96.0	24.0–154.0	72.0–199.0			
HIV-infected infants, *N*	3	2	1	2	2	2	1	1	1
Median time (IQR)	18.0	10.0	15.0	7.0	49.0	94.5	10.0	66.0	112.0
	(11.0–24.0)	(5.0–15.0)		(1.0–13.0)	(42.0–56.0)	(87.0–102.0)			
HIV-infected infants, range	11.0–24.0	5.0–15.0	—	1.0–13.0	42.0–56.0	87.0–102.0	—	—	—

^*∗*^The number of infants contributing to each step varied as some dates were not available to calculate all steps for each infant. ^+^All values are presented in days except for the number of infants contributing to each step (*N*); ART: antiretroviral therapy; IQR: interquartile range; *N*: number; NRL: National Reference Laboratory.

**Table 2:** Turnaround time (in days) for all cohort study children diagnosed as HIV-infected at birth and 6 weeks during the entire cohort study (July 2014–October 2016).

	Blood draw to receipt at NRL^+^	Receipt of specimen at NRL to testing at NRL	Testing at NRL to result receipt at health facility	Result receipt at facility to receipt by caregiver	Blood draw to receipt by caregiver	Infant age at receipt by caregiver	Result receipt by caregiver to treatment initiation	Blood draw to ART initiation	Infant age at treatment initiation
*Diagnosed by birth DNA PCR test *(*n* = 5)									
All infants, *N*^*∗*^	3	3	3	5	5	5	5	5	5
Median time (IQR)	21.0	2.0	12.0	1.0	38.0	43.0	0	38.0	45.0
	(12.0–21.0)	(0–4.0)	(12.0–16.0)	(0–4.0)	(35.0–40.0)	(41.0–45.0)	(0–1.0)	(35.0–48.0)	(43.0–49.0)
All infants, range	12.0–21.0	0–4.0	12.0–16.0	0–19.0	28.0–61.0	29.0–61.0	0–8.0	28.0–62.0	29.0–62.0

*Diagnosed by 6-week DNA PCR test *(*n* = 2)									
All infants, *N*^*∗*^	2	2	2	2	2	2	2	2	2
Median time (IQR)	30.5	7.0	8.0	4.0	49.5	97.5	6.5	56.0	104.0
	(28.0–33.0)	(6.0–8.0)	(8.0–8.0)	(3.0–5.0)	(49.0–50.0)	(93.0–102.0)	(0–13.0)	(49.0–63.0)	(102.0–106.0)

^*∗*^The number of infants contributing to each step varied as some dates were not available to calculate all steps for each infant. ^+^All values are presented in days except for the number of infants contributing to each step (*N*); ART: antiretroviral therapy; IQR: interquartile range; *N*: number; NRL: National Reference Laboratory.
